# Patient satisfaction, quality of life, and catheter-related complications in long-term urinary catheter users: a nationwide survey

**DOI:** 10.1007/s00345-025-05850-8

**Published:** 2025-08-01

**Authors:** Coen H.H. Christiaans, Felice E. E. van Veen, Jeroen R. Scheepe, Bertil F. M. Blok

**Affiliations:** Department of Urology, Erasmus MC Rotterdam, Dr. Molewaterplein 40, Rotterdam, 3015 GD The Netherlands

**Keywords:** Clean intermittent catheterization, Indwelling catheterization, Lower urinary tract symptoms, Neurogenic bladder, Patient satisfaction, Quality of life

## Abstract

**Purpose:**

To compare patient satisfaction, quality of life, catheter-related complications between three types of catheterization in long-term urinary catheter users. To improve clinical decision-making for long-term urinary catheter users.

**Methods:**

A nationwide survey study was conducted from August to September 2024. Patients who apply clean intermittent catheterization (CIC), have an urethral indwelling catheter (IDC), or a suprapubic catheter (SPC), were identified through the MediReva database, a Dutch medical supplier. The survey was developed by structured consensus meeting and consisted of the ICIq-LTCqol and the EQ-5D-5 L.

**Results:**

3320 patients participated in the study (response rate 33%). 2634 performed CIC, 383 had an IDC, and 303 had an SPC. 75.9% was male and the mean age was 72 years. CIC patients reported the best patient satisfaction and QoL scores. When corrected for multiple confounders IDC and SPC were independently associated with lower patient satisfaction and QoL scores. There was no difference in UTI incidence in the last 6 months between the groups.

**Conclusions:**

This study shows differences in patient satisfaction, QoL and, catheter-related complications between three types of catheterization. Healthcare providers should be aware of the impact of bladder drainage methods on the patient satisfaction and QoL, especially for those using an IDC or SPC. This information can be of added value in the decision-making process of long-term bladder management.

**Supplementary Information:**

The online version contains supplementary material available at 10.1007/s00345-025-05850-8.

## Introduction

Lower urinary tract dysfunction (LUTD) involves a broad spectrum of problems, ranging from bladder emptying to storage problems. The origin of LUTD can be neurogenic including conditions such as spinal cord injury (SCI), spina bifida, Parkinson’s disease and multiple sclerosis [1]. Or non-neurogenic including conditions such as bladder outlet obstruction (benign prostatic hyperplasia, urethral stricture), post-pelvic surgery retention, or idiopathic [2]. 

Both types can cause urinary retention. The primary goal is timely and complete emptying of the bladder to prevent overflow incontinence, urinary tract infections (UTI), and renal failure [3]. Clean intermittent catheterization (CIC) is considered the method of choice for bladder drainage, as it is thought to reduce the risk of catheter-associated UTIs, bladder stones, and renal deterioration compared to indwelling catheters (urethral indwelling catheters (IDC) and suprapubic catheters (SPC)) [4]. At the same time, CIC is thought to increase the quality of life (QoL) through greater independence, mobility, and maintaining the ability to engage in sexual activity [5, 6]. However, to independently perform CIC, manual dexterity, strength, and sufficient cognitive ability are required [7]. 

Indwelling catheters are indicated in those situations where there is no other reasonable choice, CIC is not possible, or the patient is not willing to perform CIC [7, 8]. IDCs are also used in the management of urinary incontinence, they can increase the QoL in selected cases of severe incontinence or when a disability makes it difficult to use the bathroom [9]. Nonetheless, the use of indwelling catheters is not without risks; it can lead to complications such as catheter blockage, discomfort (bladder spasms), urethral injury, catheter-associated UTIs and bladder stones [10]. The guidelines prefer the use of an SPC over an IDC when a patient needs an indwelling catheter for a longer period of time [4]. 

In the recent two decades the use of urinary catheters in the Netherlands has substantially increased [11, 12]. Although CIC is considered the preferred method for bladder drainage, indwelling catheters are used more often in the Netherlands [12]. Because of the aging population, the number of people using a urinary catheter is expected to grow in the future [13]. To optimize the standard of care and minimize catheter-related complications, it is essential that each patient receives the most appropriate type of bladder catheterization.

This study aims to compare the three types of bladder catheterization to identify the differences in patient satisfaction, QoL, and catheter-related complications. These factors can influence the selection which urinary catheter is the most suitable for a patient. Therefore, identifying these factors can enhance the clinical management and informed decision-making process regarding urinary catheters, ensuring choices are tailored to individual needs and circumstances of each patient.

## Materials and methods

### Patient population

A nationwide Dutch survey study was conducted from August to September 2024. Participants included in this study were patients older than 16 years of age, who performed CIC, had an IDC or SPC in the community setting for at least six months. Patients were identified though the MediReva data base. MediReva is a medical supplier which provides medical devices to patients across the Netherlands. Invitations to participate were distributed via email by MediReva. Prior to the start of the survey the patient had to give digital informed consent. All data was collected anonymously. This study was approved by the local Erasmus Medical Ethical Review Committee (MEC-2024-0303).

### Survey development

The survey was composed of two validated questionnaires and a self-made questionnaire. The patient satisfaction and QoL was evaluated with the International Consultation on Incontinence Long-Term Indwelling Catheter Users quality of life (ICIQ-LTCqol), a validated questionnaire used to evaluate the impact on QoL and outcome of treatment in long-term catheter users [14]. This questionnaire has three domains: the catheter function and concern score, lifestyle impact score, and unscored items. In this survey the questions were adapted to ensure relevance for patients performing CIC. The questionnaire was translated to Dutch by the research team. The health-related QoL was measured using the Dutch version of the Euroqol 5 Dimensional 5 Level (EQ-5D-5 L) instrument [15]. The catheter-related complications were assessed using a self-made questionnaire created by the research team, consisting of two physician researchers and two urologists, and was based on their clinical experience. UTI was defined as “urinary infections” that will make you feel unwell or require you to take antibiotics, this definition is also used in the ICIQ-LTCqol [14]. Hematuria was defined as visible blood in the urine. The survey was constructed in Castor EDC, an open source online data capture application.

### Statistical analysis

All categorical variables are presented as frequencies and percentages. Continuous variables are presented as mean and standard deviation (SD). Categorical outcomes were analyzed using chi-square tests, continuous outcomes were analyzed using the ANOVA or Kruskal-Wallis test. We used the 5%-trimmed mean to calculate the mean incidence of UTIs in the past six months, to adjust for extreme values. Multivariable regression analysis was performed for baseline variables potentially predicting the ICIQ-LTCqol and EQ-5D-5 L scores. The variables included were age, gender, BMI and, type of catheterization. In the type of catheterization group CIC was the reference category, since CIC is the preferred method for bladder emptying [4, 5]. In the gender group, male was the reference category since most of our respondents were male. Statistical analyses were performed using IBM SPSS statistics version 25 with a critical significance level of *P* < 0.05.

## Results

### Patient characteristics

In total, the survey was sent to 10,109 patients, of which 5,512 participated in the study. Patients who completed at least 50% of the questionnaire were included, resulting in a total of 3,320 participants (response rate 33%). Among these, 2634 (79.3%) performed CIC, 383 (11.5%) had an IDC, and 303 (9.1%) had an SPC. The majority of patients was male (75.9%). The relative number of men was lowest in the CIC group (*p* < 0.001). The mean age of the patients was 72 years (SD 4.82). The average age of patients in the IDC group was higher compared to the CIC and SPC group (*p* < 0.001). 19.2% of the patients had an urinary catheter because of a known neurogenic disease. The baseline characteristics are shown in Table 1.

**Table 1:** Baseline characteristics

	CIC	IDC	SPC	Total	*p*-value
Number of patients (%)	2634 (79.3)	383 (11.5)	303 (9.1)	3320 (100)	
Male (%)	1963 (74.5)	319 (83.3)	237 (78.2)	2519 (75.9)	< 0.001
Female (%)	671 (25.5)	64 (16.7)	66 (21.8)	799 (14.1)	< 0.001
Mean age, years (SD)	71 (12)	78 (10)	73 (12)	72 (12)	< 0.001
Age groups, years (%) 0–45	106 (4.0)	5 (1.3)	8 (2.6)	119 (3.6)	
45–55	162 (6.2)	7 (1.8)	18 (5.9)	187 (5.6)	
55–65	395 (15.0)	23 (6.0)	40 (13.2)	458 (13.8)	
65–75	939 (35.7)	103 (26.9)	84 (27.7)	1126 (33.9)	
75–85	900 (34.2)	159 (41.5)	117 (38.6)	1176 (35.4)	
> 85	131 (5.0)	86 (22.5)	36 (11.9)	253 (7.6)	
Mean BMI, kg/m*2 (SD)	25.95 (4.21)	27.06 (6.58)	27.36 (6.19)	26.23 (4.82)	< 0.001
Reason for catheterisation (%)					
*Neurogenic*	517 (19.6)	54 (14.1)	66 (21.8)	637 (19.2)	
*Prostate related problem*	501 (19.0)	113 (16.7)	84 (27.7)	698 (21)	
*Hypocontractile/ acontractile bladder*	789 (30)	54 (14.1)	34 (11.2)	877 (26.4)	
*Overactive bladder*	139 (5.3)	35 (9.1)	27 (8.9)	201 (6.1)	
*Incontinence*	111 (4.2)	43 (11.2)	21 (6.9)	175 (5.3)	
*Cancer related*	137 (5.2)	26 (6.8)	22 (7.3)	185 (5.6)	
*Urethral stricture*	31 (1.2)	4 (1.0)	15 (5.0)	50 (1.5)	
*Recurrent UTIs*	23 (0.9)	3 (0.8)	0	26 (0.8)	
*Interstitial cystitis/ bladder pain syndrome*	3 (0.1)	0	1 (0.3)	4 (0.1)	
*Urinary retention (unspecified)*	122 (4.6)	7 (1.8)	3 (1.0)	132 (4.0)	
*Other*	74 (2.8)	15 (3.9)	16 (5.3)	105 (3.2)	
*Unknown*	187 (7.1)	29 (7.6)	14 (4.6)	230 (6.9)	
Duration of catheter use (%)					
*6 months- 1 year*	408 (15.5)	113 (29.5)	48 (15.8)	569 (17.1)	
*1–5 years*	1348 (51.2)	209 (54.6)	168 (55.4)	1725 (52,0)	
*More than 5 years*	878 (33.3)	61 (15.9)	87 (28.7)	1026 (30.9)	

*IDC* indwelling catheter, *SPC* suprapubic catheter, *CIC* clean intermittent catheterization, *SD* standard deviation, *BMI* Body mass Index

### Catheter-related complications

The 5% trimmed mean number of UTIs over the past six months in CIC patients was 0.86 (SD 1.18), in IDC and SPC patients this was 0.82 (SD 1.13) and 0.97 (SD 1.20), respectively (*p* = 0.22). In total, 462 (13.9%) used antibiotic prophylaxis, incidence was highest in the CIC group (*p* = 0.01). The incidence of hematuria and bladder stones in the past 6 months was lower in CIC patients compared to IDC and SPC patients(for both, *p* < 0.001). Results are presented in Table 2.

**Table 2:** Catheterization efficacy

	CIC (*n* = 2634)	IDC (*n* = 383)	SPC (*n* = 303)	Total (*n* = 3320)	*P* value
Number of UTIs past 6 months, 5% trimmed mean (SD)	0.86 (1.18)	0.82 (1.13)	0.97 (1.20)		0.22
Number of UTIs grouped (%) *0*	1425 (54.1)	211 (55.1)	152 (50.2)	1788 (53.9)	
*1*	521 (19.8)	71 (18.5)	58 (19.1)	650 (19.6)	
*2*	271 (10.3)	50 (13.1)	42 (13.9)	363 (10.9)	
*3 or more*	471 (15.8)	51 (13.3)	51 (16.8)	573 (15.6)	
On antibiotic prophylaxis (%)	391 (14.8)	39 (10.2)	32 (10.6)	462 (13.9)	0.01
Type of antibiotic prophylaxis *Nitrofurantoine*	227 (58.0)	20 (51.3)	13 (40.6)	260 (56.3)	
*Trimethoprim*	53 (13.5)	8 (20.5)	3 (9.4)	64 (13.9)	
*Fosfomycine*	46 (11.7)	4 (10.3)	4 (12.5)	55 (11.9)	
*I don’t know*	11 (2.8)	1 (2.3)	3 (9.4)	15 (3.2)	
*Other*	57 (14.5)	6 (15.4)	8 (25)	71 (15.4)	
Incidence hematuria in the past 6 months (%)	626 (23.8)	183 (47.8)	152 (50.2)	961 (28.9)	< 0.001
Incidence of bladder stones in the past 6 months (%)	92 (3.5)	68 (17.8)	78 (25.7)	238 (7.2)	< 0.001

*CIC* clean intermittent catheterization, *IDC* indwelling catheter, *SPC* suprapubic catheter, *UTI* urinary tract infection, *SD* standard deviation

### ICIQ-LTCqol outcomes

#### Catheter function and concern

**Fig. 1:**
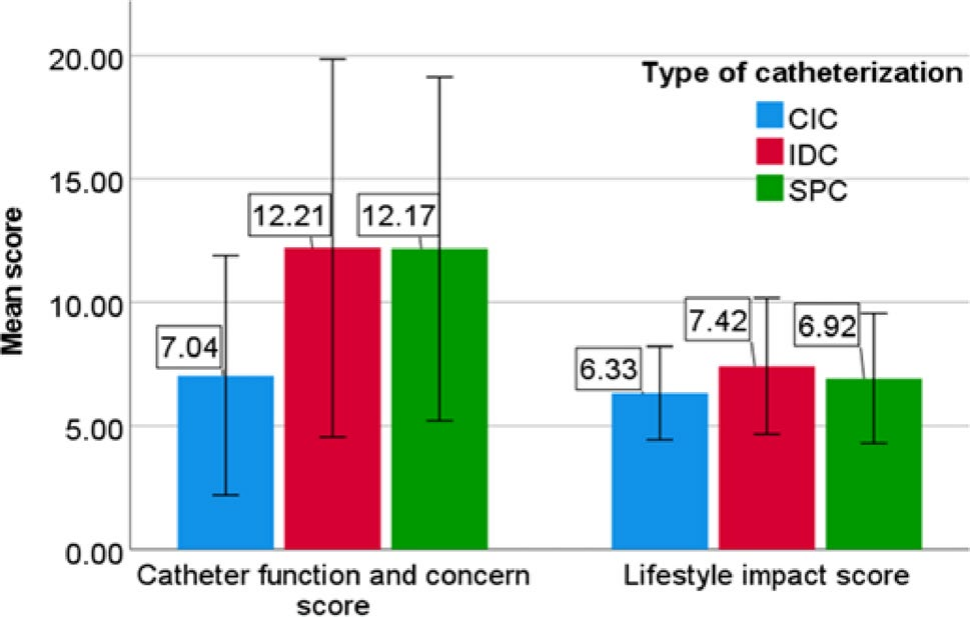
ICIQ-LTCqol: mean catheter function and concern and lifestyle impact scores. *CIC* clean intermittent catheterization, *IDC* indwelling catheter, *SPC* suprapubic catheter,

The question on which CIC users endorsed the highest levels of impact on QoL was: “How often do you have ‘urinary infections’ that make you feel unwell or require you to take antibiotics?” (n = 1696, 65%). For IDC users this question was: “Do you feel you have adapted to life with a catheter?” (n = 281, 76%). For SPC users this questions was: “Does your catheter cause you any pain, discomfort or soreness?”(n = 216, 77%). In CIC patients the catheter prevented sexual activity less (n = 510, 20.8%) compared to the IDC (n = 146, 46%) and SPC patients (n = 112, 40.2%) (p < 0.001). The mean catheter function and concern score was 7.04 (SD 4.84), 12.14 (SD 7.67), and 12.10 (SD 6.93) in CIC, IDC, and SPC patients, respectively (P < 0.001). Results are shown in supplementary Tables 1 and Fig. 1.

#### Lifestyle impact

IDC patients most often reported a negative effect of the catheter on their ability to travel (*n* = 113, 33.1%), take part in social activities (*n* = 93, 27.2%), and go out of the house (*n* = 16, 15.3%). In contrast, IDC patients also most often reported a positive effect of the catheter on their ability to go out of the house (*n* = 72, 21.7%). SPC patients most often reported a positive effect of the catheter on their ability to travel (*n* = 56, 20.1%) and take part in social activities (*n* = 54, 19.4%). In all three groups, the majority of participants reported that the catheter had no effect on their lifestyle. The mean lifestyle impact score was 6.33 (SD 1.89), 7.42 (SD 2.75), and 6.92 (SD 2.62) in CIC, IDC, and SPC patients, respectively (*p* < 0.001). Results are shown in supplementary Tables 3 and Fig. 1.

#### EQ-5D-5 L

**Fig. 2:**
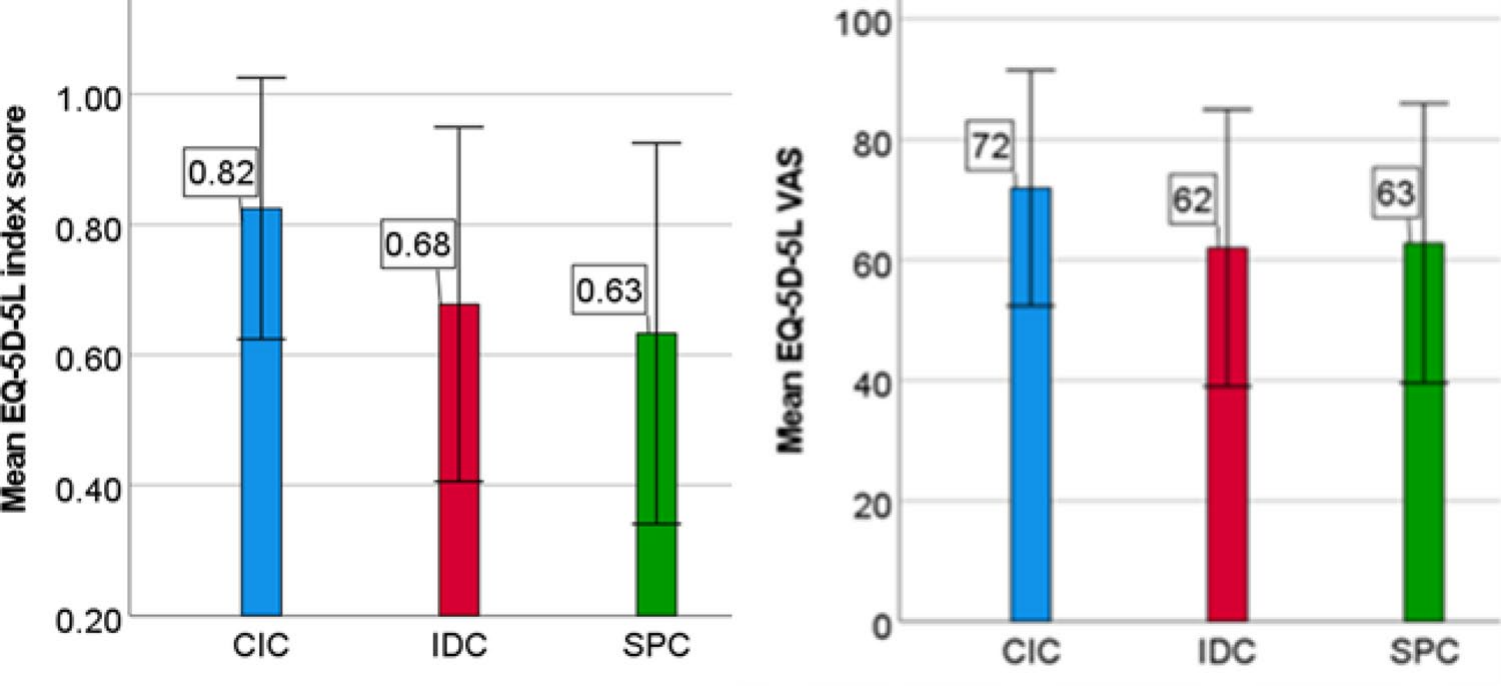
EQ-5D-5 L health value and VAS (visual analogue scale). *IDC* indwelling catheter, *SPC* suprapubic catheter, *CIC* clean intermittent catheterization

The mean EQ-5D-5 L index score was higher in CIC patients (0.82, SD 0.2) compared to IDC (0.68, SD 0.27), and SPC patients (0.63, SD 0.29) (*p* < 0.001). The mean VAS score was also higher in CIC patients (72, SD 20) compared to IDC (62, SD 23) and SPC patients (63, SD 23) (*p* < 0.001). Results are shown in supplementary Tables 1 and Fig. 2.

#### Multivariable regression analysis

Multivariable regression analysis taking into account age, gender, BMI, and type of catheterization showed that an IDC was associated with a lower catheter function and concern score (β 0.308, 95% CI 5.053–6.250), lifestyle impact score (β 0.153, CI 0.791–1.271), EQ-5D-5 L VAS (β -0.166, CI -13.307– -8.569), and EQ-5D-5 L health value (β -0.241, 95% CI -0.200– -0.152) compared to CIC. SPC was also associated with a lower catheter function and concern score (β 0.257, 95% CI = 4.565–5.869), lifestyle impact score (β 0.077, 95% CI 0.307–0.826), EQ-5D-5 L VAS (β -0.128, 95% CI -11.695– -6.627), and EQ-5D-5 L health value (β -0.241, 95% CI -0.223– -0.171) compared to CIC. Results are shown in Table 3.

**Table 3:** Multivariable regression analysis

	Catheter function and concern score			Lifestyle impact score			EQ-5D-5 L VAS			EQ-5D-5 L health value		
	Mean	β	95% CI	Mean	β	95% CI	Mean	β	95% CI	Mean	β	95% CI
Gender												
*Male*	7.7	Ref	Ref	6.54	Ref	Ref	72	Ref	Ref	0.82	Ref	Ref
*Female*	9.31	**0.087**	0.72–1.64	6.39	-0.006	-0.21–0.15	64	**-0.132**	8.25– -4.64	0.70	**-0.172**	-0.112– -0.075
Age (years)	–	**-0.152**	0.09– -0.06	–	**0.046**	0.00–0.01	–	**0.062**	0.04–0.18	–	**0.190**	0.003–0.004
BMI (kg/m*2)	–	**0.042**	0.01–0.09	–	-0.003	-0.17–0.15	–	**-0.064**	-0.44– -0.13	–	**-0.102**	-0.007– -0.003
Catheter-type												
*CIC*	7.06	Ref	Ref	6.33	Ref	Ref	72	Ref	Ref	0.82	Ref	Ref
*IDC*	12.14	**0.308**	5.05–6.25	7.42	**0.153**	0.79–1.27	62	**-0.166**	-13.30– -8.57	0.68	**-0.241**	-0.200– -0.152
*SPC*	12.10	**0.257**	4.57–5.87	6.92	**0.077**	0.31–0.83	63	**-0.128**	-11.70– -6.63	0.63	**-0.248**	-0.223– -0.171

*IDC* indwelling catheter, *SPC* suprapubic catheter, *CIC* clean intermittent catheterization, *VAS* visual analog scale, *β* Beta coefficient, *CI* Confidence Interval

Bold numbers are statistically significant (*P* < 0.05)

## Discussion

Urinary catheters are a commonly used device in the treatment of neurogenic and non-neurogenic LUTD. In the present study, most patients performed CIC and the majority was male. CIC patients had the lowest scores in the ICIQ-LTCqol, indicating a less negative impact of the catheter on their QoL. Additionally, CIC patients had the highest scores in de EQ-5D-5 L, indicating CIC patients perceive their QoL to be better compared to IDC and SPC patients. Multivariable regression analysis showed IDC and SPC were both independently associated with lower ICIQ-LTCqol and EQ-5D-5 L scores.

Consistent with the literature urinary catheters were more frequently used with increasing age [12]. However, the group aged over 85 years was significantly smaller. A possible explanation is that individuals in this age group have less access to email, and consequently, to our survey. Additionally, this age group is more likely to be residing in a nursing home, which prevented them from participating in our study. Consistent with the literature, younger patients are more likely to use CIC, whereas older patients tend to utilize indwelling catheteres more frequently [12]. 

One previous study reported on the prevalence of neurogenic and non-neurogenic causes for urinary catheter users in the Netherlands [11]. In this study, 20.7% of participants had a neurogenic cause; however, due to a registration error, the underlying condition could not be determined for 34.4% of the participants. Therefore, there could be a possible underestimation of (non-)neurogenic bladder population. In our study, 19.2% reported a neurogenic cause for catheter usage. However, a considerable part reported “Urinary retention (unspecified)” or “Unknown” as the cause for their urinary catheter. This may indicate, that also in our study, an underestimation of the (non-)neurogenic bladder population happened. Therefore, caution is necessary for interpretation and generalizability of these results.

Our study found no significant difference in UTIs in the past 6 months between the three groups. A recent study comparing UTI frequency in 500 CIC and IDC patients also found no significant difference between the groups in the past 12 months [16]. This is in contrast to the EAU guidelines, which state that CIC has a lower risk of UTI compared to IDC and SPC [4]. This difference can be due to the different definitions used. Because of the design of this study, we were unable to diagnose UTIs through urine cultures.

In line with the literature, our study reported a lower incidence of hematuria and bladder stones the past 6 months in CIC patients compared to IDC and SPC patients [17, 18]. 

There is one prior study that uses the ICIQ-LTCQoL questionnaire to assess the QoL in long-term urinary catheter users. Youssef et al., included 141 patients > 18 years of with an indwelling catheter in Egypt [19]. The average function and concern score for IDC (19.00 vs. 12.14) and SPC patients (19.97 vs. 12.10) and the average lifestyle impact score in IDC (9.95 vs. 6.92) and SPC patients (10.1 vs. 7.42) were higher compared to our study. These differences may be attributed to Egypt’s high burden of disease and the Netherlands’ higher standard of care [19, 20]. 

Multiple studies have compared the patient QoL between different bladder emptying methods in patients with neurogenic lower urinary tract dysfunction (NLUTD) [21–23]. The patients included in these studies are different from our study. In this study the majority reported a non-neurogenic cause for their LUTD. Therefore, comparative analysis between these studies should be performed with caution.

Akkoç et al. included 195 cases with traumatic SCI and assessed the differences in QoL using the King’s Health Questionnaire [21]. They found no difference in QoL between the CIC group and to the IDC group. Sekido et al. reported on 282 participants with SCI and used the Qualiveen30 and EQ-5D-5 L questionnaires [22]. There was no significant difference in QoL between the CIC and IDC group. The EQ-5D-5 L health value was significantly higher in the CIC group compared to the IDC group, consistent with our study. The discrepancy in QoL between the CIC and IDC groups in these studies and our study can be attributed to the fact that patients with SCI can be wheelchair bounded, tetraplegic, or have reduced dexterity. These factors can strongly influence or even determine the likelihood of successful CIC performance and their satisfaction and QoL. Furthermore, changes in internal standards due to the neurogenic disease, namely changes in their concept of QoL and personal expectations over time may account for improved satisfaction despite using less than ideal urinary management options [22]. 

In a Dutch cohort Adriaansen et al. performed a survey study in 242 Dutch individuals with SCI using the short-form Qualiveen questionnaire [23]. In accordance with the findings in our study, CIC is the most frequently used method for bladder emptying and patients who use an indwelling catheter (IDC or SPC) to empty the bladder reported a lower QoL compared to patients performing CIC.

Multivariable regression analysis showed that the type of catheterization is independently associated with the patient satisfaction and QoL scores. IDC and SPC had the largest association of the included variables with lower outcomes in both ICIQ-LTCqol and EQ-5D-5 L. However, the different analyses only explained 16% (catheter function and concern score), 3% (lifestyle score), 19% (EQ-5D-5 L health value), and 7% (EQ-5D-5 L VAS) of the variance of the total scores. This shows that patient satisfaction and QoL is influenced by many other factors than we have included, such as psychological factors (i.e. adjustment, coping, self-esteem), financial circumstances, and social support [24]. 

An indwelling catheter is indicated when there is no other reasonable choice, CIC is not possible, or the patient is not willing to perform CIC [7]. In clinical practice, IDCs are often used without meeting appropriate indications or are retained longer than strictly necessary [25, 26]. The EAU guideline advice an SPC over an IDC when a long-term urinary catheter is indicated, because of the complications associated with an IDC, especially the damage it can cause to the penile and bulbar urethra [4]. In this study, more patients used an IDC compared to an SPC. Given that all users are long-term users, this is in contrast to the recommendations of the EAU guidelines [4]. Where suprapubic catheterization may be considered as the best option if CIC is not possible, in practice, IDC is often the initial approach because the procedure can be organized and managed by nursing staff. Whereas initial placement of a SPC requires an urological procedure. Thereby, SPC has contraindications which do not apply to urethral catheterization, such as bladder carcinoma, prior major abdominal surgery, skin infection and coagulopathy [27]. 

Although CIC is the preferred method of catheterization, there are still some problems accompanied to the technique. First, some patients change from CIC to indwelling catheters because of inconvenience, high BMI, urinary leakage, and UTIs [28–30]. Secondly, studies show that long-term adherence to CIC can be a challenge [31, 32]. Because of these reasons, providing comprehensive guidance during the initiation of CIC and ensuring regular follow-up are important [33]. Al Hasan et al., showed that a patient-centered chronic care self-management program was associated with fewer hospital visits and higher adherence in the first month [34]. 

Future studies should focus on decreasing the environmental impact of CIC [35]. To reduce the plastic waste, reusable catheters could be a solution. Thereby, when using reusable catheters, CIC will become more available in low income countries where single use catheters are at present too expensive for the healthcare system [36]. 

This study has several limitations. First, this study did not use a validated questionnaire for both indwelling catheters and self-catheterization to assess the patient satisfaction and QoL, due to the lack of such a questionnaire.

Secondly, there was no validated Dutch translation of the ICIQ-LTCqol available.

Thirdly, a limitation of survey studies is inclusion bias. Patients with more severe catheter problems may have enrolled at a higher rate to express their problems or in the hope of learning more about their bladder management.

However, the present study has several strengths. To date this is the largest questionnaire study on long- term bladder management in non-neurogenic and neurogenic patients. Secondly, this study included patients with non-neurogenic, whereas prior studies primarily focused on neurogenic patients. Giving that the majority of patients who use urinary catheters have a non-neurogenic disease, which differ substantially from neurogenic patients, this is an important population to include in research about bladder management.

## Conclusion

Our results indicate that healthcare providers should be aware of the impact of bladder-emptying methods on the patient satisfaction and QoL in long-term catheter users. In patients with adequate dexterity CIC should always be discussed, emphasizing the increased QoL and decreased catheter-related complications. If a patient is unwilling to perform CIC, comprehensive counseling that addresses potential catheter-related complications and the impact on QoL associated with indwelling catheters may help overcome both internal and external barriers to perform CIC.

**Statements and Declarations**.

## Supplementary Information

Below is the link to the electronic supplementary material.

Supplementary Material 1

## Data Availability

The data that support the findings of this study are not openly available due to reasons of sensitivity and are available from the corresponding author upon reasonable request.
