# Supplementary material for: Patient satisfaction, quality of life, and catheter-related complications in long-term urinary catheter users: a nationwide survey
Source: World J Urol. 2025 Aug 1;43(1):470. doi: 10.1007/s00345-025-05850-8 (PMC12316781; doi:10.1007/s00345-025-05850-8)
Supplement: Supplementary file 1 — Supplementary Material 1 [file 345_2025_5850_MOESM1_ESM.docx]

| Supplementary tables: | CIC (n=2634) | IDC (n=383) | SPC (n=303) | P value |
| --- | --- | --- | --- | --- |
| Is the possibility of leaking on your mind? (%) |  |  |  | <0.001 |
| *Never* | 1909 (72.3) | 111 (29.0) | 115 (38.2) |  |
| *Occasionally* | 428 (16.3) | 104 (27.2) | 93 (30.9) |  |
| *Sometimes* | 246 (9.3) | 95 (24.8) | 57 (18.9) |  |
| *Most of the time* | 33 (1.3) | 41 (10.7) | 19 (6.3) |  |
| *All of the time* | 23 (0.9) | 32 (8.4) | 17 (5.6) |  |
| How problematic is your catheter? (%) |  |  |  | <0.001 |
| *No problems* | 1932 (73.4) | 138 (36.2) | 97 (32.0) |  |
| *Some problems but rather keep it* | 368 (14.0) | 76 (19.9) | 58 (19.1) |  |
| *Some problems but have to keep it* | 311 (11.8) | 139 (36.5) | 127 (41.9) |  |
| *Lots of problems* | 21 (0.8) | 28 (7.3) | 21 (6.9) |  |
| How often do you have 'urine infections' that make you feel unwell or require to take antibiotics? (%) | | | | 0.961 |
| *Never* | 923 (35.2) | 141 (37.3) | 105 (34.9) |  |
| *Less than once a year* | 919 (35.1) | 129 (34.1) | 97 (32.2) |  |
| *A couple of times a year* | 534 (20.4) | 70 (18.5) | 66 (21.6) |  |
| *Once every 2-3 months* | 138 (5.3) | 21 (5.6) | 18 (6.0) |  |
| *About once a month* | 71 (2.7) | 11 (2.9) | 10 (3.3) |  |
| *Several times per month* | 34 (1.3) | 6 (1.6) | 6 (2.0) |  |
| Do you feel you have adapted to life with a catheter? (%) |  |  |  | <0.001 |
| *Completely* | 930 (35.7) | 89 (24.1) | 88 (29.5) |  |
| *Mostly* | 730 (28.0) | 122 (33.0) | 87 (29.2) |  |
| *Somewhat* | 376 (14.4) | 92 (24.9) | 71 (23.8) |  |
| *Not really* | 328 (12.6) | 49 (13.2) | 38 (12.8) |  |
| *Not at all* | 240 (9.2) | 18 (4.9) | 14 (4.7) |  |
| Does your catheter cause you any pain, discomfort or soreness? (%) | |  |  | <0.001 |
| *Never* | 1074 (43.9) | 93 (27.3) | 63 (22.6) |  |
| *Occassionally* | 978 (40.0) | 110 (32.3) | 110 (39.4) |  |
| *Sometimes* | 310 (12.7) | 90 (26.4) | 54 (19.4) |  |
| *Most of the time* | 68 (2.8) | 27 (7.9) | 35 (12.5) |  |
| *All of the time* | 16 (0.7) | 21 (6.2) | 17 (6.1) |  |
| Does having a catheter prevent sexual activity? (%) |  |  |  | <0.001 |
| *Never* | 30 (5.6) | 28 (10.1) | 1033 (42.3) |  |
| *Occassionally* | 11 (3.2) | 15 (5.4) | 180 (7.4) |  |
| *Sometimes* | 9 (2.6) | 18 (6.5) | 155 (6.3) |  |
| *Most of the time* | 30 (8.8) | 21 (7.6) | 85 (3.5) |  |
| *All of the time* | 96 (28.2) | 58 (20.9) | 90 (3.7) |  |
| *Not applicable* | 136 (40.0) | 115 (41.4) | 762 (31.2) |  |
| *Don't wish to answer* | 28 (8.2) | 23 (8.3) | 136 (5.6) |  |
| Mean catheter function and concern score (SD) | 7.06 (4.84) | 12.14 (7.67) | 12.10 (6.93) | <0.001 |

**Supplementary table 1.** ICIQ-LTCqol: questions that endorsed the highest impact on QoL and the average catheter function and concern score. IDC (indwelling catheter), SPC (suprapubic catheter), CIC (clean intermittent catheterization), SD (standard deviation).

|  | CIC (n=2634) | IDC (n=383) | SPC (n=303) | P value | |
| --- | --- | --- | --- | --- | --- |
| Does your catheter affect your ability to travel? (%) |  |  |  | <0.001 |  |
| *The catheter has helped my ability to travel.* | 399 (16.2) | 50 (14.7) | 56 (20.1) |  |  |
| *The catheter has no effect on my ability to travel* | 1278 (51.8) | 109 (32) | 90 (32.3) |  |  |
| *The catheter limits my ability to travel* | 572 (23.2) | 98 (28.7) | 79 (28.3) |  |  |
| *I don't travel because of my catheter* | 33 (1.3) | 15 (4.4) | 11 (3.9) |  |  |
| *I don't travel bur for other reasons* | 183 (7.4) | 69 (20.2) | 43 (15.4) |  |  |
| Does your catheter affect your social activities? (%) |  |  |  | <0.001 |  |
| *The catheter has helped my ability to take part in social activeties* | 394 (16.0) | 55 (16.1) | 54 (19.4) |  |  |
| *The catheter has no effect on my social activities* | 1627 (66.2) | 159 (46.5) | 140 (50.2) |  |  |
| *The catheter limits my ability to take part in social activities* | 343 (13.9) | 79 (23.1) | 65 (23.3) |  |  |
| *I don't take part in social activities because of my catheter* | 22 (0.9) | 14 (4.1) | 1 (0.4) |  |  |
| *I don't take part in social activities but for other reasons* | 73 (3.0) | 35 (10.2) | 19 (6.8) |  |  |
| Does your catheter affect your ability to go out of the house? (%) |  |  |  | <0.001 |  |
| *The catheter has helped me to go out* | 428 (17.4) | 72 (21.7) | 57 (20.4) |  |  |
| *The catheter has no effect on my ability to go out* | 1824 (74.3) | 193 (56.6) | 178 (63.8) |  |  |
| *The catheter limits my ability to go out* | 184 (7.5) | 51 (15.0) | 31 (11.1) |  |  |
| *I don't go out because of my catheter* | 2 (0.1) | 1 (0.3) | 0 (0) |  |  |
| *I don't go out but for other reasons* | 18 (0.7) | 22 (6.5) | 13 (4.7) |  |  |
| Mean lifestyle impact score (SD) | 6.33 (1.89) | 7.42 (2.75) | 6.92 (2.62) | <0.001 |  |

**Supplementary table 2.**  ICIQ-LTCqol: questions and mean lifestyle impact score. IDC (indwelling catheter), SPC (suprapubic catheter), CIC (clean intermittent catheterization), SD (standard deviation).

|  | CIC (n=2634) | IDC n=383 | SPC (n=303) | Total (n=3320) | P value |
| --- | --- | --- | --- | --- | --- |
| EQ-5D-5L index score, mean (SD) | 0.82 (0.20) | 0.68 (0.27) | 0.63 (0.29) | 0.79 (0.23) | <0.001 |
| EQ-5D-5L VAS, mean (SD) | 72 (20) | 62 (23) | 63 (23) | 70 (21) | <0.001 |

**Supplementary table 3.** EQ-5D-5L health value and VAS. IDC (indwelling catheter), SPC (suprapubic catheter), CIC (clean intermittent catheterization), VAS (visual analog scale), SD (standard deviation).
